# THE EFFECT OF INTERVENTIONS TO REDUCE SOCIAL ISOLATION AND LONELINESS AMONG FAMILY CAREGIVERS: A SYSTEMATIC REVIEW

**DOI:** 10.1093/geroni/igad104.0622

**Published:** 2023-12-21

**Authors:** Boah Kim, Lun Li, Andrew Wister

**Affiliations:** Simon Fraser University, Vancouver, British Columbia, Canada; MacEwan University, Edmonton, Alberta, Canada; Simon Fraser University, Vancouver, British Columbia, Canada

## Abstract

Social isolation and loneliness are among the key risk factors for deleterious health and well-being among family caregivers. Interventions to reduce caregiver isolation and loneliness are of great interest as family caregivers are essential in community care, particularly senior care. The current study aims to review the literature on interventions regarding social isolation and loneliness among family caregivers and summarize successful strategies for reducing isolation and loneliness among family caregivers. A systematic literature review was conducted. Electronic databases were searched, and 1130 peer-reviewed journal articles published in English since 2000 were identified. A total of 34 eligible studies (16 quantitative studies, 8 qualitative studies, and 10 mixed methods studies) were included in this review. Findings from qualitative studies (including qualitative components from mixed methods studies) consistently reveal the positive experience of family caregivers in reducing isolation and loneliness. However, findings from quantitative studies add more layers of complexity with considerable evidence of failing to identify the change of isolation or loneliness after attending interventions. The studied caregiver groups, length of intervention, and scales used to measure isolation and loneliness are closely relevant to the effect of the intervention. Besides peer support, mentoring and skill training, technology (Internet-based programs) plays a significant role in facilitating family caregivers to maintain social interaction and connectedness. This review confirms the pleasant impacts of different interventions in reducing caregiver isolation and loneliness, but also calls for more research in identifying changes of isolation and loneliness both short- and long-term among family caregivers.

